# Analysis of the accuracy and reliability of vertical jump evaluation using a low-cost acquisition system

**DOI:** 10.1186/s13102-023-00718-z

**Published:** 2023-09-06

**Authors:** Luis C. Caseiro-Filho, Carlos E. Girasol, Mateus L. Rinaldi, Tenysson W. Lemos, Rinaldo R. J. Guirro

**Affiliations:** Department of Health Sciences, Ribeirão Preto Medical School of the University of São Paulo (USP) - Ribeirão Preto, São Paulo, Brazil

**Keywords:** Video analyses, Biomechanics, Free software, ICC, Athletic performance

## Abstract

**Background:**

The vertical jump can be analyzed based on the flight time achieved by the individual. This measurement can be obtained using a force platform or a three-dimensional infrared camera system, but such equipment is expensive and requires training for data collection and processing. Thus, this study aimed to evaluate the accuracy and reliability of using a smartphone and the Kinovea software compared with a force platform as a method of vertical jump analysis.

**Methods:**

For this purpose, two independent evaluators analyzed videos of bipodal and unipodal vertical jumps by counting the variables among participants. The participants performed three consecutive jumps in bipodal and unipodal conditions with the dominant and non-dominant legs.

**Results:**

The intra-rater analysis for bipodal jumps was found to have excellent reproducibility (ICC = 0.903 to 0.934), whereas for unipodal jumps, the reproducibility was moderate to excellent (ICC = 0.713 to 0.902). The inter-rater analysis showed that for bipodal jumps, the reproducibility is substantial to excellent (ICC = 0.823 to 0.926), while for unipodal jumps, it is moderate (ICC = 0.554 to 0.702).

**Conclusions:**

Therefore, it can be concluded that the vertical jump evaluation can be performed using the smartphone-Kinovea system. However, the same evaluator should carry out the evaluation to maintain reliable indices.

## Introduction

Recent literature highlights the vertical jump as an important assessment tool for variables such as lower limb power [1, 2], analysis of peripheral fatigue [3, 4], and domains of biomechanics to improve an athlete’s performance [3, 5]. Among the indices presented in an evaluation of the vertical jump, the value of flight time stands out [6, 7]commonly employed in clinical and scientific practice, as a reliable variable during the task. The vertical jump assessment can also be used to monitor the external load imposed in a workout, thus underlining the importance of applying its method. After volunteers took consecutive jumps, a calculation using the height reached and the impulse time was made so evaluators could be able to manage the training load of the participant based on these indices [8].

A recent systematic review [9] has provided data on the importance of other variables besides flight height that can be analyzed for performance evaluation. Indices such as power, peak velocity, peak force, and average impulse were highlighted. In this regard, considering the number of variables that the vertical jump can generate, many studies seek to find ways to evaluate it reliably. Thus, the use of a force platform for data acquisition stands out, currently being the gold standard of this evaluation. However, it imposes limitations given its cost and difficulty in transporting it from one site to another for data collection. A three-dimensional infrared camera system [10] and contact platforms [11] have been used as alternative methods. However, such resources still show access limitations due to their cost, transportation constraints, and apart from their dependence on electricity.

The high cost of the platforms and the limitations imposed on their daily application end up interfering with their use in different environments. The difficulty that professionals face in conducting vertical jump tests as evaluative means in environments outside standardized laboratories should be noted. As a matter of fact, cheaper methods have been used to evaluate jump performance, such as the Sargent test [12] and some research groups have assessed vertical jump using other tools, such as a specific phone app [13], associated with practical sports models and machine-learning scenarios [14], or even associated with different remarkable accessories to the gesture, such as the inclusion of specific shoes [15]. Studies such as [16] sought more cost-effective alternatives and used high-speed capture cameras to measure maximum vertical height and flight time with open-access software (Kinovea), observing high reliability and reproducibility when comparing data from an infrared platform and the captured images. However, although high-speed cameras are less expensive when compared to force platforms, professionals with fewer resources still need help to afford to use them. In this way, low-cost analysis models are a remarkable combination. As mentioned, tools such as the Kinovea software have been widely explored in sport practice scenarios, and the wide dissemination of smartphones around the world has configured an environment of noticeable insertion and considerable low cost when compared to other assessment instruments. Thus, this study aimed to evaluate the accuracy and the intra- and inter-rater reliability of maximum vertical height, impulse time, and flight time using open-access software (Kinovea) with video captured by a smartphone.

## Methods

### Subjects

This study was approved by the Ethics Committee of the Clinical Hospital of the Ribeirão Preto Medical School under protocol 4.188.366. The participants were recruited through social media and with posters scattered around the university campus. The research was conducted at the Laboratory of Physiotherapeutic Resources (LARF) of the Ribeirão Preto Medical School of the University of São Paulo (FMRP-USP), and the evaluation time was set according to the participant’s availability to come to the collection site.

To be included, individuals should present or report: age between 18 and 40 years old, male gender, absence of musculoskeletal lesions in lower limbs and trunk in the last three months, and the lack of cardiovascular diseases. As for the exclusion criteria, the authors established that the volunteers would be excluded if they could not perform the vertical jump tests. Previous training was not offered since the study compares only the outcomes presented, using two methods and their relationship. The study was designed to analyze the Intraclass Correlation Coefficient (ICC) values of the explored variables based on a mean-rating (k = 2), absolute-agreement, 2-way mixed-effects model. The number of instruments was considered equal to 2, expected ICC of 0.7, a confidence interval amplitude for of ICC of 0.3, and a confidence coefficient of 95% [17]. A sample size of 46 volunteers was obtained, considering a minimum sample size associated with a possible sample loss of 10%.

### Experimental approach to the problem

The present study conducted a correlation analysis between the data obtained through the evaluation of the jump using a force platform and the Kinovea software, as well as the accuracy, reliability, and reproducibility of this intra- and inter-rater analysis. The variables analyzed were: flight time, impulse time, and maximum height reached.

The data was collected by an experienced rater, who also conducted the data analysis on the force platform. Two other trained raters, with prior experience in video analysis and Kinovea software manipulation, performed the analysis. The jumps were made in the following order: bipodal jump, unipodal jump using the dominant limb, and unipodal jump using the non-dominant limb.

### Instrumentation

A force platform (AMTI OR6-7, Watertown, MA, USA) and a smartphone (Motorola Moto X4, Chicago, IL, USA) were used to collect the data on vertical jumps. The smartphone camera recordings were analyzed using the openly licensed Kinovea software (Kinovea 0.8.15 for Windows, available at http://www.kinovea.org). The force platform had an area of 50 × 50 cm, where it analyzed forces in the mediolateral (ML), anteroposterior (AP), and vertical (V) directions, as well as the moments of forces around these axes. A sampling frequency of 200 Hz was imposed, and a MiniAmp MSA-6 amplifier, AMTI (Advanced Mechanical Technology, Inc.), was used. The data were obtained using BioDynamics-BR software (Biodynamics-BR1—DataHominis Technology).

For smartphone recording, the device had a sampling frequency of 30 fps, 12 MP, and video recording at 2160 p. The camera was positioned at a sufficient frontal point to capture the movement with the focus stabilized, where the synchronization of the frames occurred through events during the jump (moment of contact with the ground, either leaving or returning to it). Two moments of the vertical jump were analyzed: the flight and impulse times. The analysis through Kinovea was measured in milliseconds. The jump height was calculated using the formula described by Glatthorn et al. [18]: $h=t^2\times1.22625$*,* where *h* is height and* t* is flight time.

### Vertical jumps performance

The bipodal and unipodal vertical jump techniques used Vertical Repetitive (cyclic) jump without the aid of the upper limbs, following the method described by Maulder and Cronin [19]. The jumps were performed on the force platform while simultaneously conducting the recordings on the smartphone for further analysis in the Kinovea software. The volunteers were instructed to flex their knees approximately at an angle of 120° and to take continuous vertical jumps at maximum effort without pauses between jumps during the tests. The trunk should be upright without excessive anteriorization, and the knees in extension during the flight phase. The test consisted of a series of three repetitions for each gesture, starting with a bilateral support jump, followed by a 60-s interval between each series [20]. For the unipodal jump, the order of dominant and non-dominant lower limbs was respected. It is important to note that the volunteers were familiarized with the vertical jump protocol before the execution of the test properly. Thus, the execution and its specificities, such as knee angle, were reinforced at this moment. It should also be pointed out that close values were indicated and did not constitute an exclusion of the volunteer.

### Flight time and impulse analysis

The analysis using the Kinovea software was conducted by two independent evaluators, with the outcomes of flight time and impulse time, as well as height achieved, to validate the accuracy and reproducibility of the method according to the Guidelines by Reporting Reliability and Agreement Studies – GRRAS [21]. The evaluators performed two analyses 15 days apart, thus making intra- and inter-examiner reproducibility measures possible [22]. The second analysis was conducted exactly as the first, where the raters took the same criteria previously imposed as the key point for analyzing the video.

Markings were made by ground contact events, either leaving or regaining contact. For flight time, the observers selected the first frame in which both feet had dropped out of ground contact until the first frame, where at least one of the feet had regained contact. For impulse time, the observers considered the onset of hip flexion as the initial event until one of the feet ceased to have contact with the ground. It should be noted that the time was obtained by the software Timer tool.

The BioDynamics Analysis software and an owner routine developed with MatLab R2015a software (The MathWorks Inc., Natick, MA) were used for the force platform analysis.

### Statistical analyses

For the statistical analysis, ICC (Intraclass Correlation Coefficient) was used to determine the intra- and inter-rater reproducibility, with the respective 95% confidence intervals, standard error of measurement (SEM), and minimum detectable change (MDC) to complement the interpretation of measurement method errors.

The interpretation of the ICC values was based on a study by Fleiss [17]: reproducibility was considered low for values below 0.40; moderate between 0.40 and 0.75; substantial between 0.75 and 0.90; and excellent above 0.90.

The data distribution was initially observed through the Kolmogorov–Smirnov test to analyze the relationship between the variables. The Pearson (r) or Spearman (rs) correlation coefficient was then applied to verify the association among the variables studied, depending on their distribution. The classification established by Munro [23] was used to interpret the magnitude of the correlations: 0.26 to 0.49, weak; 0.50 to 0.69, moderate; 0.70 to 0.89, high; and 0.90 to 1.00, very high. Cronbach’s alpha was used to analyze the reliability of the observed measures. Its value ranges from 0 to 1, and values closer to 1 indicate that the values measure the same dimension. Statistical processing was performed using SPSS® software, version 20.0 (Chicago, IL, USA).

## Results

A total of 46 participants were recruited, with a mean age of 24.65 ± 3.80 years, a weight of 85 ± 16.90 kg, a height of 1.76 ± 0.07 m, and a body mass index of 27.25 ± 4.61 kg/m^2^. The values for the vertical jump evaluation can be found in Table 1.

**Table 1:** Impulse time, flight time, and maximum vertical jump height

	**Outcomes**	**Force Platform**		**Video analysis**					
				**Rater 1**				**Rater 2**	
				**Test**		**Retest**		**Test**	
		**Mean (SD)**	**CI 95%**	**Mean (SD)**	**CI 95%**	**Mean (SD)**	**CI 95%**	**Mean (SD)**	**CI 95%**
Bipodal	Impulse time (s)	0.85 (0.28)	0.79, 0.92	0.74 (0.20)	0.69, 0.78	0.71 (0.19)	0.67, 0.75	0.67 (0.19)	0.63, 0.72
	Flight time (s)	0.47 (0.08)	0.45, 0.49	0.49 (0.05)	0.48, 0.50	0.49 (0.05)	0.48, 0.50	0.45 (0.05)	0.44, 0.47
	Maximum vertical height (cm)	29 (1.00)	26, 31	29 (0.6)	28, 31	30 (0.6)	29, 31	26 (0.6)	24, 27
Dominant	Impulse time (s)	1.09 (0.44)	0.93, 1.25	0.81 (0.25)	0.75, 0.87	0.78 (0.23)	0.72, 0.83	0.67 (0.19)	0.62, 0.71
	Flight time (s)	0.33 (0.54)	0.31, 0.35	0.34 (0.05)	0.33, 0.35	0.36 (0.04)	0.35, 0.37	0.31 (0.04)	0.30, 0.32
	Maximum vertical height (cm)	14 (0.4)	12, 15	15 (0.4)	14, 16	16 (0.4)	15, 17	12 (0.3)	11, 12
Non-Dominant	Impulse time (s)	1.03 (0.37)	0.90, 1.17	0.78 (0.21)	0.74, 0.83	0.73 (0.21)	0.69, 0.78	0.63 (0.19)	0.59, 0.67
	Flight time (s)	0.35 (0.08)	0.32, 0.38	0.34 (0.05)	0.33, 0.35	0.35 (0.04)	0.34, 0.36	0.31 (0.06)	0.30, 0.33
	Maximum vertical height (cm)	16 (0.7)	13, 18	15 (0.4)	14, 15	15 (0.4)	14, 16	12 (0.5)	11, 13

*SD* standard deviation, *CI 95* confidence interval 95%, *s* seconds, *cm* centimeters

The intra-rater reproducibility for bipodal jumping showed ICC values ranging from 0.903 to 0.934 between impulse time, flight time, and maximum height, thus demonstrating excellent reproducibility. For the unipodal dominant and unipodal non-dominant conditions, it was observed that ICC values ranged from 0.713 to 0.950 and 0.874 to 0.902, respectively. Thus, moderate to excellent reproducibility was observed for these conditions. The complete values with SEM and MDC indices can be seen in Table 2.

**Table 2:** Intra-rater reliability of vertical jump indices

**Outcomes**	**α**	**ICC**	**95% CI**	**SEM (°)**	**SEM (%)**	**MDC (°)**
**Rater 1**						
**Bipodal**						
Impulse time (s)	0.903	0.898	0.840, 0.935	0.04	5.95	0.12
Flight time (s)	0.928	0.925	0.884, 0.952	0.01	1.96	0.03
Maximum vertical height (cm)	0.934	0.932	0.893, 0.956	0.02	0.05	0.04
**Dominant**						
Impulse time (s)	0.950	0.946	0.909, 0.967	0.04	5.44	0.12
Flight time (s)	0.738	0.716	0.537, 0.823	0.02	5.33	0.05
Maximum vertical height (cm)	0.713	0.691	0.500, 0.807	0.02	0.14	0.06
**Non-Dominant**						
Impulse time (s)	0.902	0.889	0.805, 0.934	0.09	8.93	0.24
Flight time (s)	0.866	0.864	0.786, 0.913	0.01	1.84	0.02
Maximum vertical height (cm)	0.874	0.872	0.799, 0.918	0.03	0.10	0.07

*α* Cronbach’s alpha, *ICC* Intra-class correlation coefficient, *CI* Confidence interval, *SEM* Standard error of Measurement, *MDC* Minimum detectable change, *s* seconds, *cm* centimeter

The inter-rater analysis for the bipodal jump showed substantial to excellent reproducibility, with values between 0.823 and 0.926. For the dominant unipodal jump, moderate reproducibility (0.684 to 0.702) and the non-dominant unipodal jump, with moderate indices (0.554 to 0.690). The values can be seen in full in Table 3.

**Table 3:** Inter-rater reliability of vertical jump indices

**Outcomes**	**α**	**ICC**	**95% CI**	**SEM (°)**	**SEM (%)**	**MDC (°)**
**Rater 1 *vs* Rater 2**						
**Bipodal**						
Impulse time (s)	0.823	0.799	0.651, 0.879	0.06	7.95	0.16
Flight time (s)	0.924	0.833	0.051, 0.943	0.01	2.61	0.03
Maximum vertical height (cm)	0.926	0.839	0.073, 0.945	0.02	0.09	0.07
**Dominant**						
Impulse time (s)	0.684	0.602	0.222, 0.779	0.11	14.92	0.31
Flight time (s)	0.702	0.572	0.022, 0.787	0.02	6.04	0.05
Maximum vertical height (cm)	0.671	0.541	0.014, 0.763	0.02	0.18	0.07
**Non-Dominant**						
Impulse time (s)	0.690	0.581	0.106, 0.781	0.16	17.12	0.45
Flight time (s)	0.641	0.579	0.265, 0.750	0.02	4.06	0.04
Maximum vertical height (cm)	0.554	0.512	0.221, 0.692	0.05	0.19	0.14

*α* Cronbach’s alpha, *ICC* Intra-class correlation coefficient, *CI* Confidence interval, *SEM* Standard error of Measurement, *MDC* Minimum detectable change, *s* seconds, *cm* centimeter

Higher ICC was thus found in the intra-rater reliability compared to the inter-rater analyses, and lower error percentages (SEM %) were found in the intra-rater reliability.

The validity of the metrics can be considered when compared to the correlation values between force platform and video jump analysis, where in the bipodal condition, a high correlation was observed for impulse time and flight time. Compared to unipodal gestures, a low correlation is shown between the methods, with only flight time and impulse time for the dominant limb showing moderate correlation, as well as for height with the non-dominant limb. All indices can be seen in Table 4.

**Table 4:** Correlation between force platform indices and video analysis of vertical jump

Outcomes	VA – Impulse time	VA – Flight time	VA – Maximum vertical height
Bipodal			
FP – Impulse time	r_s_ 0.838, *p* 0.00^*^	-	-
FP – Flight time	-	r_s_ 0.894, *p* 0.00^*^	-
FP – Maximum vertical height	-	-	r_s_ 0.575, *p* > 0.99
Dominant			
FP – Impulse time	r_s_ 0.527, *p* 0.00^*^	-	-
FP – Flight time	-	r 0.511, *p* 0.00^*^	-
FP – Maximum vertical height	-	-	r_s_ 0.127, *p* 0.489
Non-Dominant			
FP – Impulse time	r -0.084, *p* 0.654	-	-
FP – Flight time	-	r 0.770, *p* > 0.99	-
FP – Maximum vertical height	-	-	r 0.500, *p* 0.00^*^

*FP* force platform, *VA* video analysis, *r* Pearson correlation coefficient, *rs* Spearman correlation coefficient, *p* p-value

^*^p < 0.05

**Fig. 1:**
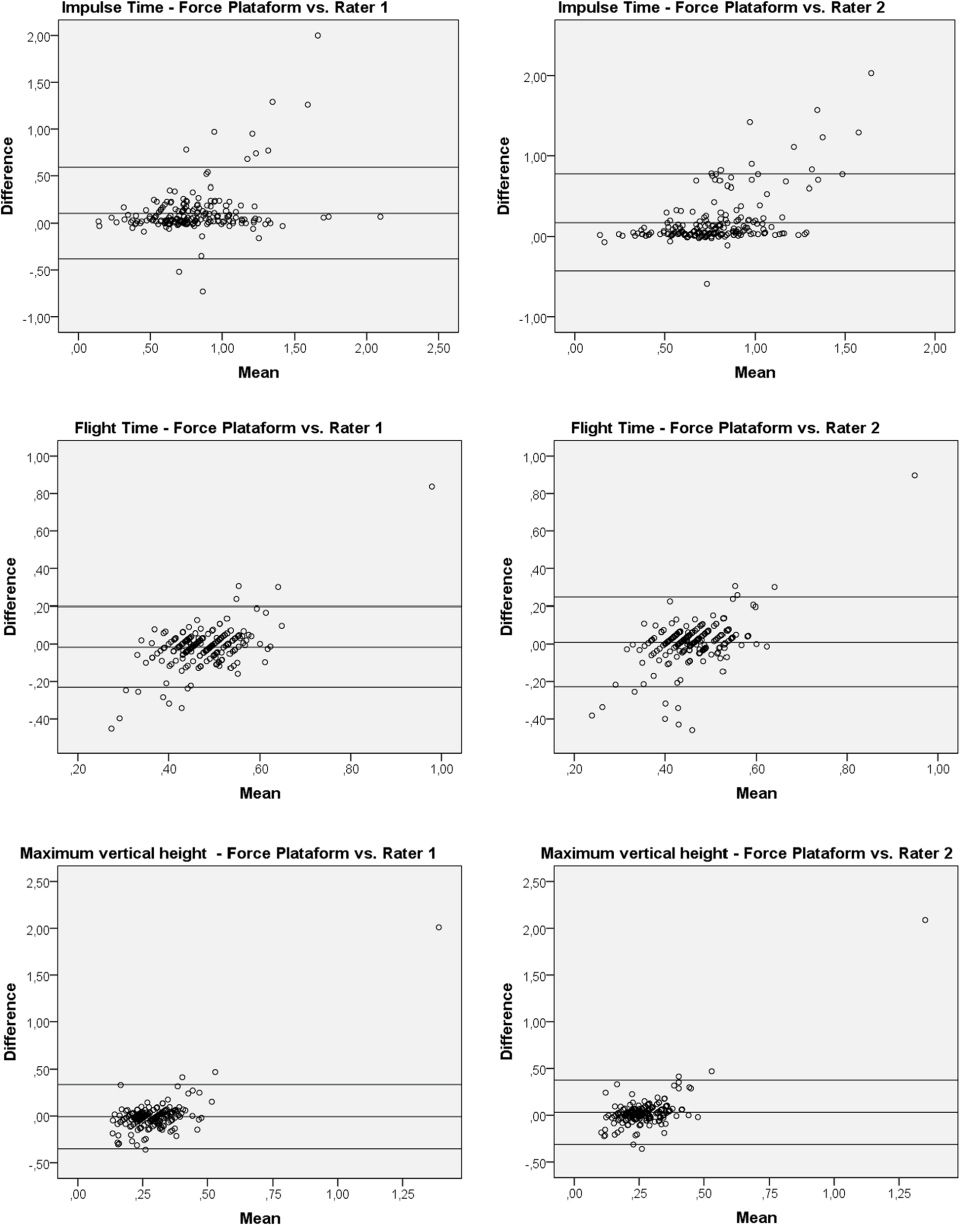
Bland–Altman analysis plots for the bipodal jumps

**Fig. 2:**
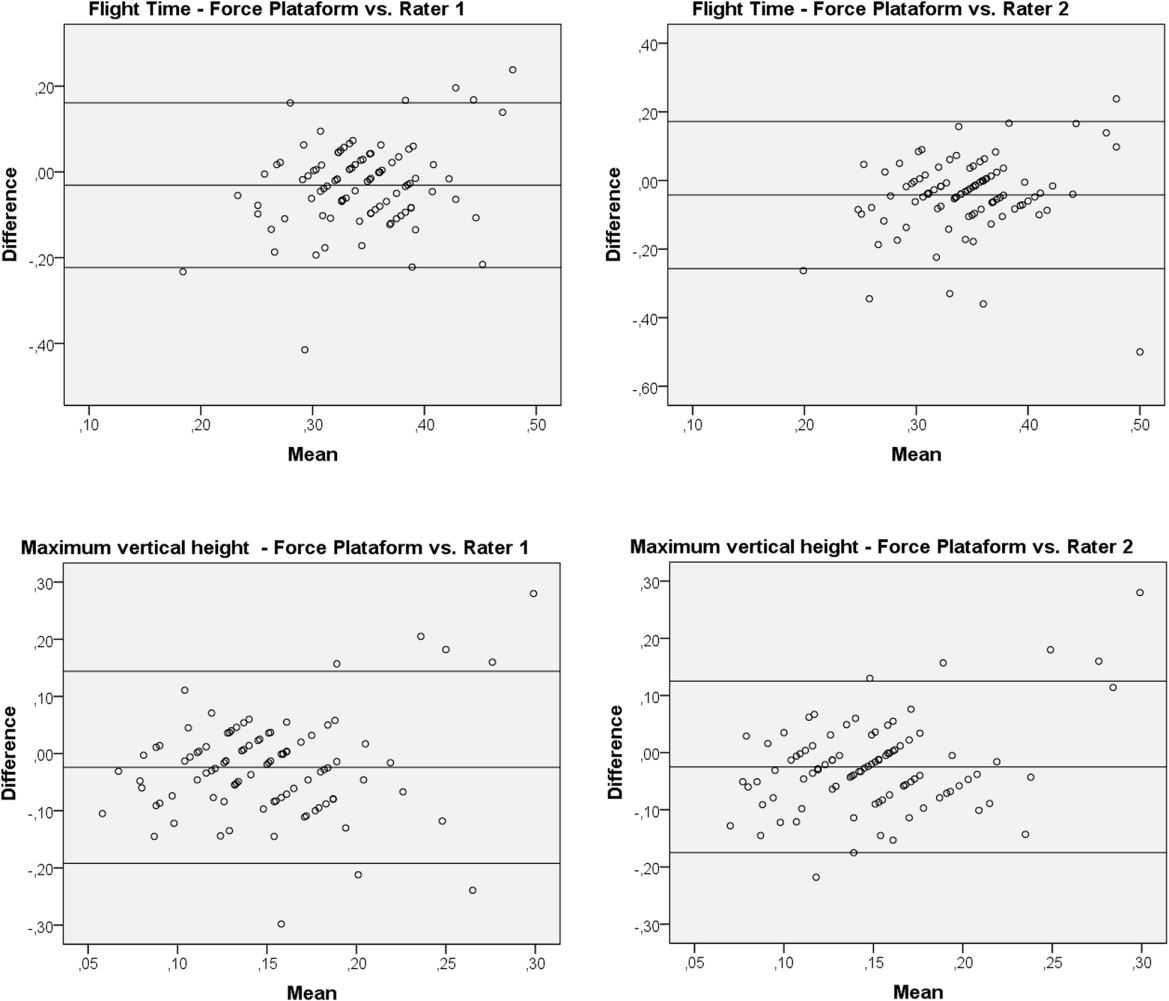
Bland–Altman analysis plots for the jumps with the dominant leg

**Fig. 3:**
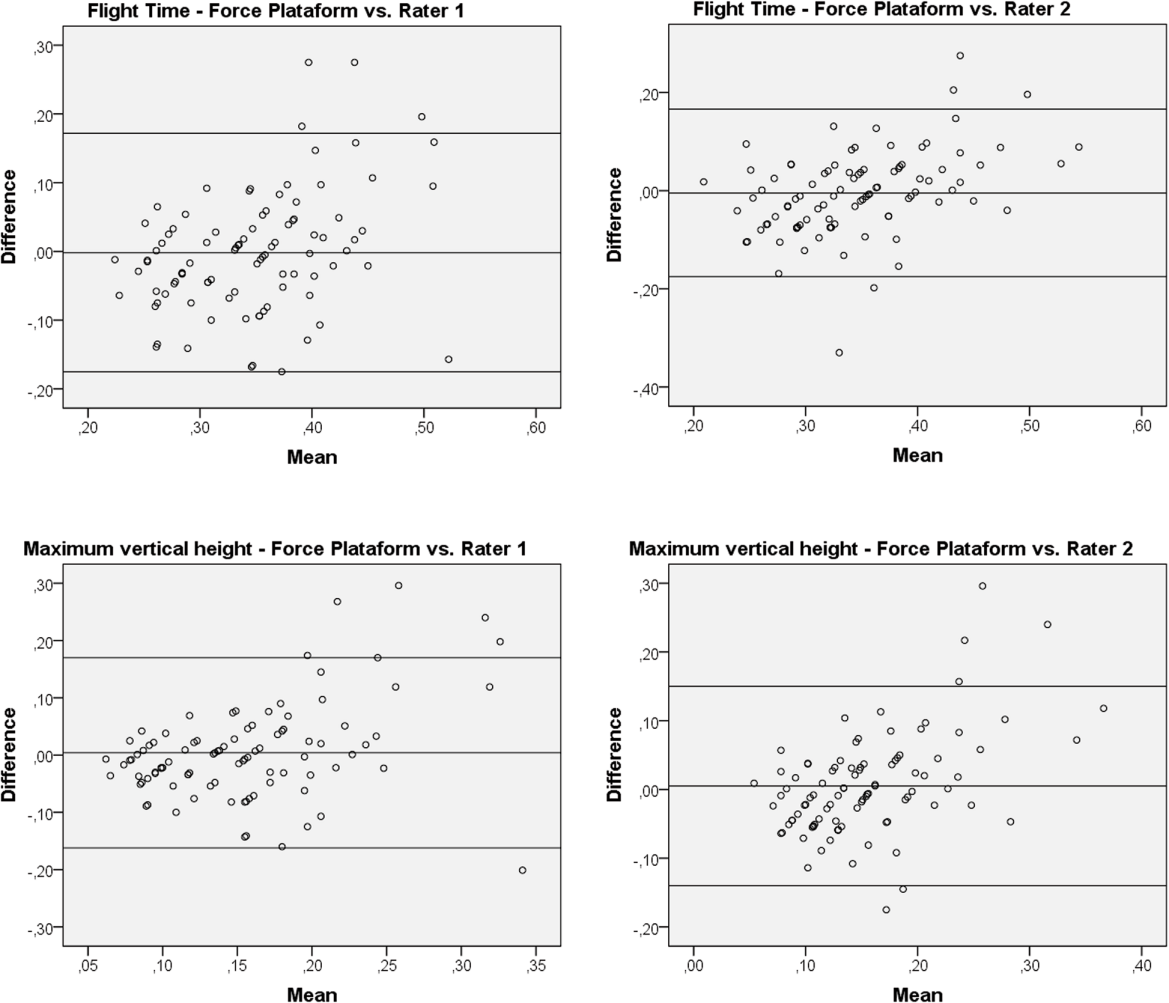
Bland–Altman analysis plots for Jumps with non-dominant leg

In Figs. 1, 2, and 3 are exposed the graphs by Bland–Altman analysis regarding the bipodal and unipodal evaluations. The graphs corroborate the figures presented previously showing high correlation between the platform and the evaluators in bipodal jumps and a low in unipodal jumps.

## Discussion

In light of the need for assertive evaluations and reliable methods, we can observe a great data discrepancy among evaluators after analyzing the present study. However, when the intra-rater data is analyzed, it is possible to notice a greater reproducibility, especially during flight time. It is possible, consequently, to say that the videos analyzed in the Kinovea software can result in reproducible evaluations in the scope of flight time, which is the most used variable for evaluative methods [6, 7]. In this regard, the importance of the current study is based on the possibility of facilitating the process of vertical jump evaluation for various purposes already presented in the literature.

As initially highlighted, the search for evaluation methods for indices, such as the vertical jump height, considering the flight time and how some intervention can improve this performance, is shown to be not only necessary but also likely to be disseminated from the financial perspective. The outcome presented in this study showed that the videos could be performed using an ordinary smartphone and should always be analyzed by the same evaluator, with a certain degree of training, to present more reliable results.

These results are in line with the literature that points out that there is a vast preference for the use of smartphones and tablets in sports practice [24], either for the quality of applications or ease of use when compared to more robust software. However, not all the tools have been submitted to scientific methods for confirmation and validation of their service, as pointed out by another study of this group [25].

Due to the importance of the vertical jump evaluation, studies seek to find more accessible means for its analysis. The study by Balsalobre-Fernandez et al. [16] evaluated the movement of the jump using a high-speed camera and demonstrated reliable results when analyzed by two evaluators. Accordingly, the data presented here corroborate Balsalobre-Fernandez’s group and add important data to the literature since a common smartphone was used and showed reliable results.

Carlos-Vivas et al. [26] analyzed the time-of-flight correlation between a cell phone app and the force platform. The results showed high reliability, demonstrating that a low-cost device can present similar results to a more robust piece of equipment. These results confirm what was observed in the current study, where the images captured by the cell phone and analyzed in the Kinovea software showed a high correlation between flight time and impulse time for bipodal jumps. It should be noted, however, that only the flight time variable had been correlated, unlike our study, which included impulse time and maximum vertical height.

The vertical jump height was evaluated in the study by Pueo, Penichet-Tomas, and Jimenez-Olmedo [27], which compared the flight height measured by a high-cost system (Motion Capture System, composed of 8 infrared cameras) using a low-cost system (Smartphone-Kinovea). The results showed high reliability between both systems. In this regard, it reiterates the capability of low-cost front-end systems as a high-quality tool. It shows, therefore, the undeniable need for the information offered by the inclusion of new technologies into the evaluation environment, where the role of science is to filter and make such tools even better, as demonstrated by Loturco et al. [28].

Points of emphasis, such as reproducibility, can be discussed regarding the tool and the proposed gesture. Rodríguez-Rosell et al. [29] conducted an essay on the reproducibility between traditional and sport-specific gestures, demonstrating that among them, the countermovement jump, such as the one used here, can be considered a reliable and reproducible gesture of the evaluated demands, thus ratifying the proposed method. Still, regarding reproducibility, a point to be highlighted is the low rates among evaluators, especially for flight time. An important point is the take-off phase of the jump, as pointed out by Mackala et al. [30]. Different positions or the noticeable change between bipodal and unipodal may be enough to promote significant changes. It is important to underline that, once it is a study of validity between the acquisition methods, the participant’s gesture did not suffer feedback or specific biomechanical demands, which could be a factor of difference in the outcome.

A recent study [31] compared the use of video at different frame rates for jump height analysis. The study aimed to analyze whether ultra-high video speed would increase the accuracy of the analysis. The researchers concluded that videos with 240 Hz were sufficient as they did not show any relevant differences compared to videos with higher frame rates. Although the current recommendation for analyzing video jumps is for videos with higher frame rates, our study presents relevant results because even with a small frame rate, we still found data that allows the use of our method for, for example, bipodal jumps. Consequently, the present study can add to the current state of the art that videos recorded by smartphones, when analyzed with the Kinovea software, can be useful for the evaluation of the vertical jump in particular instances, with excellent reproducibility when measured by the same evaluator, and consistent values for clinical practice. Based on the fact that low-cost analysis models are a remarkable combination, the scenario of simple and ordinary acquisition and open-access software creates an environment of remarkable insertion and considerable low cost.

This study shows some limitations, such as the difficulty in standardizing key moments for biomechanical and gesture analysis. However, the proposal of the present study is based on the validity of the evaluative means. Moreover, the study only included healthy male individuals. Future studies should evaluate individuals of both genders with some dysfunction that may alter the analyzed gesture, and larger time windows may allow a more assertive analysis, for example.

## Conclusion

The current study demonstrates that time-of-flight analyses of bipodal jumps can be performed using a smartphone-Kinovea system. Nevertheless, it should be noted that the use of this system is more reliable when analyzed by a single evaluator. For analysis of unipodal jumps, the present study did not observe data supporting the use of a smartphone for movement analysis.

## Data Availability

All data are available in the manuscript. If necessary, the corresponding author can make the data available for consultation.
